# Emergence of the GII-4 Norovirus Sydney2012 Strain in England, Winter 2012–2013

**DOI:** 10.1371/journal.pone.0088978

**Published:** 2014-02-13

**Authors:** David J. Allen, Natalie L. Adams, Farah Aladin, John P. Harris, David W. G. Brown

**Affiliations:** 1 Virus Reference Department, Microbiology Services, Public Health England, London, United Kingdom; 2 Gastrointestinal, Emerging and Zoonotic Infections Department, Heath Protection Services, Public Health England, London, United Kingdom; 3 Institute of Infection and Global Health and National Consortium for Zoonosis Research, University of Liverpool, Liverpool, United Kingdom; National Institute of Allergy and Infectious Diseases, United States of America

## Abstract

Norovirus is the commonest cause of acute gastrointestinal disease and is the main aetiological agent of outbreaks of gastroenteritis, particularly in semi-closed environments. Norovirus infections in England typically peak between December and March each year. The most commonly detected norovirus strains belong to the genetically diverse genogroup-II genotype-4 (GII-4) genocluster and in the previous two norovirus winter seasons the majority of GII-4 strains in circulation worldwide have been genetically similar to the GII-4 strain New Orleans 1805/2009/USA. At the beginning of the 2012/13 season a genetically distinct GII-4 strain (Sydney 2012/NSW0514/2012/AU) was described which emerged worldwide during the winter of 2012/13. Here we describe the emergence of norovirus strains genetically related to Sydney2012 in England during the 2012/13 season to replace NewOrleans2009 strains as the most commonly detected variant of GII-4 norovirus in England. Furthermore, we demonstrate that whilst the emergence of Sydney2012 coincided with an early peak in the number of norovirus outbreaks, there was not an overall increase in norovirus activity compared to the previous season. Finally, we show that the Sydney2012 strain is associated with distinct genetic changes compared to the NewOrleans2009 strain, and these changes may have contributed to the emergence of the Sydney2012 strain.

## Introduction

Noroviruses are the commonest cause of acute gastrointestinal disease in England and Wales [1], [2] and is recognised as the main aetiological agent of outbreaks of infectious intestinal disease (IID). Outbreaks are associated with semi-closed environments and have the greatest impact and occur with the highest frequency in healthcare-associated settings, particularly hospitals and care homes [3]. Norovirus infections are associated with the winter months [4], and a typical norovirus season runs September–April, with a peak in numbers of outbreaks between December and March each year.

*Norovirus* is a single genus of the *Caliciviridae* family of positive-sense single-stranded RNA viruses. The *Norovirus* genome is approximately 7500 nucleotides in length, and is organised as three open reading frames (ORFs). The 5′-ORF (ORF1) encodes a large polyprotein that undergoes post-translational processing into the viral non-structural proteins; ORF2 encodes the major capsid protein, VP1; and ORF3 encodes a small basic protein (VP2) which is thought to be a minor structural protein.

A number of functional domains have been identified in the VP1 protein [5]: the shell (S) domain forms the contiguous shell of the virus, whilst the protruding (P) domain forms the surface-exposed spike structures on the virus surface. The P domain is further sub-divided into P1 and P2, and it is the P2 domain that represents the most surface-exposed area of the virus capsid. The P2 domain is genetically hypervariable and has been associated with virus-host interactions including cell attachment via histo-blood group antigens (HBGAs) and it is also thought to be the site of neutralising antibody epitopes [6]–[8].

The *Norovirus* genus shows high genetic variability, and classification of the *Norovirus* genus as five recognised genogroups (GI-GV) is based on the sequence diversity in the ORF2-encodded VP1 protein [9]. The majority of human norovirus strains belong to genogroup I (GI) and genogroup II (GII), and these are further divided into multiple genotypes/genoclusters [9]. The most commonly detected norovirus strains belong to the genetically diverse genogroup-II genotype-4 (GII-4) genocluster. The GII-4 strains are most commonly detected in association with outbreaks in healthcare settings [10]. Strain replacement events occur periodically among circulating GII-4 norovirus strains [11]–[13], and can coincide with higher than average levels of norovirus activity. Strain replacement events leading to increased levels of norovirus activity have been observed in England in the winter seasons of 2001/02 [14], 2005/06 and 2009/10 [15].

In the previous two norovirus winter seasons (2010/11 & 2011/12), the majority of GII-4 strains in circulation worldwide have been genetically similar to the GII-4 strain New Orleans 1805/2009/USA (Accession Number: GU445325) [16]. At the beginning of the 2012/13 season (September-December 2012), international public health agencies reported the emergence of a GII-4 strain (Sydney 2012/NSW0514/2012/AU, Accession Number: JX459908) that is genetically distinct from those previously detected in circulation [17].

Here we describe the emergence of norovirus strains genetically related to JX459908/Sydney 2012/NSW0514/2012/AU (hereafter referred to as Sydney2012) in England during the 2012/13 season to replace strains genetically similar to GU445325/New Orleans 1805/2009/USA (hereafter referred to as NewOrleans2009) as the most commonly detected GII-4 norovirus genetic cluster in England. Furthermore, we demonstrate that the emergence of Sydney2012 was associated with an early peak in the number of norovirus outbreaks, and that the Sydney2012 strain is associated with distinct genetic changes compared to the NewOrleans2009 strain.

## Materials and Methods

### Data Sources

#### Laboratory surveillance of norovirus in England

In the period May 2012 to June 2013, 592 faecal samples from 592 norovirus-confirmed outbreaks were referred to the Virus Reference Department, Public Health England, as part of a sentinel norovirus strain surveillance programme in England. Referrals were collated from geographically disparate regions in England.

Briefly, suspected norovirus outbreaks are defined locally if there are two or more cases in a functional care unit within a hospital that present: (a) two or more episodes of vomiting of suspected infectious cause occurring in a 24 hour period; or (b) two or more loose stools in a 24 hour period; or (c) one or more episodes of both vomiting and diarrhoea within a 24 hour period. Laboratory testing would be performed on faecal specimens from at least two symptomatic patients from the care unit. Where at least two norovirus-positive laboratory results are obtained, a single norovirus-positive sample would be referred for strain characterisation as part of the sentinel norovirus strain surveillance programme.

#### The Hospital Norovirus Outbreak Reporting System

The Hospital Norovirus Outbreak Reporting System (HNORS; www.hpa-bioinformatics.org.uk/noroOBK/) was introduced in January 2009 by the former Health Protection Agency in conjunction with the Infection Prevention Society in order to collect information on the epidemiology of norovirus outbreaks in hospitals in England. HNORS is a web-based surveillance system whereby infection control staff based in hospitals are able to enter summary information on outbreaks of suspected or confirmed norovirus in hospitals. This standard dataset collects key epidemiological information on each outbreak including dates of first and last onset, number of patients and staff affected, whether there were any ward or bay closures and ward type. Monthly counts (May 2012 to June 2013) of laboratory confirmed outbreak reports were extracted from HNORS for use in analyses.

### Laboratory Techniques

#### Nucleic acid extraction and reverse transcription

Faecal samples were prepared as 10% suspensions in a balanced salt solution (Modified Eagles Medium, Gibco, Life Technologies, Paisley, UK). Total nucleic acid was extracted from a 200 µl aliquot of faecal suspension using the QIAxtractor automated nucleic acid extraction platform (QIAGEN Ltd., Crawley, UK), VX Reagent & Plasticware kit (QIAGEN). Complementary DNA (cDNA) was generated by reverse transcription as follows: a 40 µl aliquot of extracted nucleic acid was heat denatured at 95°C for 5 minutes then quenched on ice. Denatured nucleic acid was mixed with 30 µl reverse transcription reaction mix, prepared as follows: 40 mM random hexamers (Invitrogen, Paisley, UK), 10 mM Tris, pH 8.0, 50 mM HCl, 5 mM MgCl_2_, 1 mM of each dNTP (Invitrogen), 400 U of MuMLV reverse transcriptase (Invitrogen). Reverse transcription was performed at 37°C for 1 hour, and the reaction was terminated by incubation at 95°C for 5 minutes, and then cooled on ice.

#### Standard genotyping analysis of norovirus strains

Outbreak samples were confirmed norovirus-positive in a genogroup-specific real-time PCR assay as previously described [18]. Genotypes were determined by partial capsid (ORF2) sequencing (region C), as previously described [19], [20].

#### Strain characterisation of GII-4 norovirus by analysis of the P2 domain

The P2 domain of GII-4 norovirus strains was amplified by polymerase chain reaction (PCR) as previously described [11], [21]. Full-length P2 domain amplicons were sequenced using a dideoxy chain-terminator method, and sequence analysis was performed using Bionumerics v6.1 (Applied Maths, Kortijk,Belgium) and MEGA version 5 [22]. Amino acid sequences were deduced from nucleotide. Throughout, amino acid motifs are designated here by standard IUPAC single-letter amino acid code.

### Modelling

#### Homology modelling of GII-4 norovirus P2 domain sequences

Amino acid residues were localised on the crystal structure of GII-4 norovirus VA387 [6] using NOC 3.1 software (http://noch.sourceforge.net/).

#### Statistical modelling of laboratory and HNORS data

A quasi-Poisson regression generalised linear model (GLM) was fitted to estimate the number of HNORS outbreak reports that could be attributed to Sydney2012. Quasi-Poisson regression is an adaptation of Poisson regression appropriate for use in the analysis of count data as it adjusts for over-dispersion of the data, where variance is greater than the mean. The model included an intercept in addition to the regression variables; this would provide a constant term to account for genogroups not explained by the variation in laboratory reporting. As norovirus is a seasonal pathogen, we included a dummy variable (winter) to account for increased number of outbreaks in wintertime.

In the initial full model, monthly HNORS reports were modelled as a function of strain-typed laboratory data and included all strain types plus the constant term and the winter dummy variable. Variables were removed if they were not significant in the model (p>0.05) or if the coefficient was negative (because this would imply negative outbreaks which is not logical). The estimated number of outbreaks attributed to Sydney2012 was calculated by multiplying the exponent of the coefficient for Sydney2012 by the number of outbreaks from HNORS. Models were fitted using R statistical software (R Core Team. R: A language and environment for statistical computing. R Foundation for Statistical Computing, Vienna, Austria 2012. http://www.R-project.org/).

## Results

### Outbreak reporting of norovirus-associated gastroenteritis in 2012/13 was associated with a shift in seasonality, but not an increase in number of outbreaks

**Figure 1:**
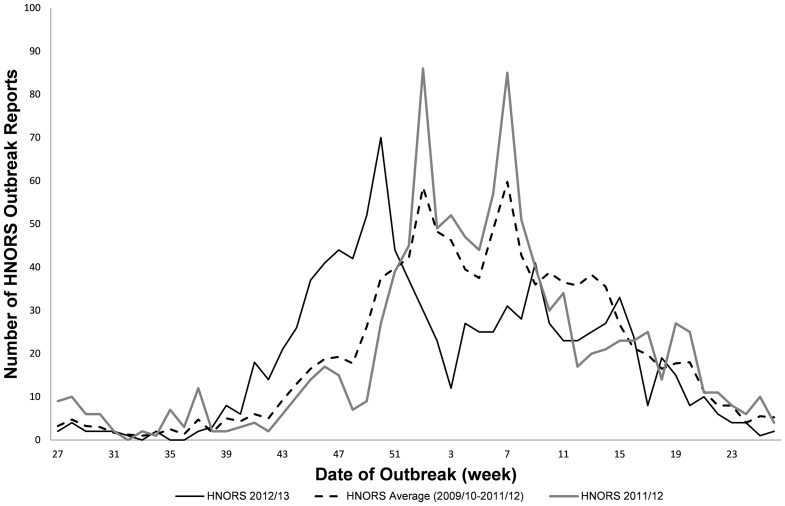
Comparison of the average number of weekly reports from HNORS for the 2009-12 seasons versus 2012/13 season in England. The solid black line indicates number of HNORS laboratory confirmed reports received in the 2012/13 season. The dashed black line indicates average number of HNORS laboratory confirmed reports received across the 2009/10 to 2011/12 seasons. The solid grey line indicates the number of HNORS laboratory confirmed reports received in the 2011/12 season.

In the early part of the 2012/13 norovirus season, between July and December 2012 (weeks 27 2012 to 52 2012) there were 480 laboratory-confirmed outbreaks reported to HNORS. This activity was 85% higher than during the same time period in 2011 (Figure 1). HNORS reports began to decline from week 50 2012, around four to eight weeks earlier than in previous seasons and the overall number of outbreaks reported to HNORS in the 2012/13 season (week 27 2012 to week 26 2013) was 9% lower than the 2011/12 season.

### Laboratory surveillance shows strains genetically similar to Sydney2012 were the most frequently detected in England throughout the 2012/13 season

A total of 592 norovirus-confirmed outbreaks occurring in England between May 2012 and June 2013 (weeks 21 2012 to 26 2013). We included outbreaks from May and June 2012 in the laboratory analysis to determine which norovirus strains were most associated with outbreaks at the end of the 2011/12 season, and leading into the 2012/13 season. The majority of these outbreaks (65.4%, n = 387/592) occurred in healthcare-associated settings, and the most frequently detected norovirus genotype was GII-4 (82%, 485/592) (Table 1), consistent with previous norovirus seasons.

**Table 1:** Distribution of norovirus strains (by genotype) in England during the 2012/13 season.

		Setting													
	Total Outbreaks	Hospital/Care Home		Community		Foodborne/Restaurant		Hotel/Holiday Resort		Cruise Ship		Other		Data Not Supplied	
**GI-3**	12 (2.0)	6	(1)	4	(0.7)		(0)		(0)		(0)		(0)	2	(0.3)
**GI-4**	13 (2.2)	3	(0.5)	2	(0.3)		(0)	1	(0.2)		(0)		(0)	7	(1.2)
**GI-6**	9 (1.5)	2	(0.3)	2	(0.3)		(0)		(0)		(0)		(0)	5	(0.8)
**GI-7**	5 (0.8)		(0)	1	(0.2)		(0)		(0)		(0)		(0)	4	(0.7)
**GI-SaitamaT35a**	8 (1.4)	2	(0.3)	1	(0.2)		(0)		(0)		(0)		(0)	5	(0.8)
**GII-1**	15 (2.5)	8	(1.4)		(0)	1	(0.2)		(0)		(0)		(0)	6	(1)
**GII-2**	2 (0.3)		(0)		(0)		(0)		(0)		(0)		(0)	2	(0.3)
**GII-3**	16 (2.7)	9	(1.5)	3	(0.5)		(0)		(0)		(0)		(0)	4	(0.7)
**GII-4/New Orleans**	135 (22.8)	91	(15.4)	3	(0.5)		(0)		(0)	2	(0.3)	1	(0.2)	38	(6.4)
**GII-4/Sydney2012**	334 (56.4)	246	(41.6)	16	(2.7)	1	(0.2)	3	(0.5)	1	(0.2)		(0)	67	(11.3)
**GII-4/Untyped**	16 (2.7)	10	(1.7)		(0)		(0)		(0)		(0)		(0)	6	(1)
**GII-5**	2 (0.3)	1	(0.2)		(0)		(0)		(0)		(0)		(0)	1	(0.2)
**GII-6**	4 (0.7)		(0)		(0)	1	(0.2)		(0)		(0)		(0)	3	(0.5)
**GII-7**	18 (3.0)	9	(1.5)		(0)		(0)		(0)		(0)		(0)	9	(1.5)
**GII-ut**	3 (0.5)		(0)	1	(0.2)		(0)		(0)		(0)		(0)	2	(0.3)
**Total**	592	387	(65.4)	33	(5.6)	3	(0.5)	4	(0.7)	3	(0.5)	1	(0.2)	161	(27.2)

(##) Per cent of all norovirus-confirmed outbreaks (*n* = 592).

Following the description of the emerging GII-4 strain Sydney2012 in December 2012 [17], the diversity among circulating GII-4 norovirus genotypes was further characterised by analysis of the hypervariable P2 domain of the capsid-encoding ORF2. To validate our analysis, 60 GII-4 norovirus-confirmed outbreaks were chosen at random from week 21 2012 to week 11 2013 of the 2012/13 season, and deduced amino acid sequences were phylogenetically compared with reference strains representing historic epidemiologically significant GII-4 genetic variants.

**Figure 2:**
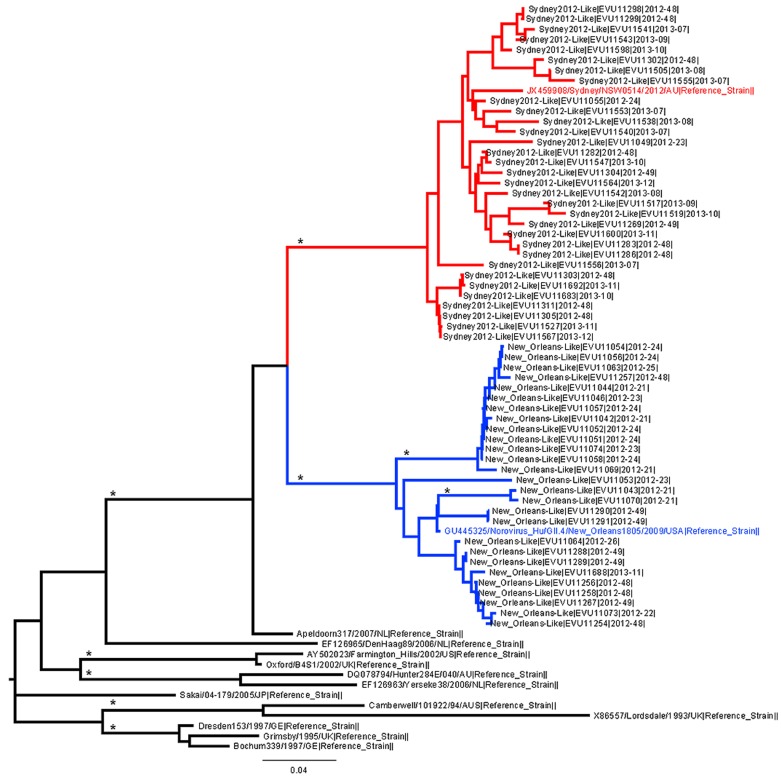
Neighbour-joining tree of 60 GII-4 norovirus P2 domain amino acid sequences detected during the 2012–2013 norovirus season in England. Red branches indicate the Sydney2012-like strains (reference strain JX459908 highlighted in red text), and blue branches indicate New Orleans 2009-like strains (reference strain GU445325 highlighted in blue text). Black branches show other epidemiologically significant GII-4 norovirus reference strains. Branches marked * have bootstrap values ≥97% after 1000 replicates. Strains sequenced as part of this study are labelled at the nodes as [Strain Type|OutbreakID|Date of Outbreak (YYYY-WW)].

Phylogenetic reconstruction using a neighbour-joining method indicated two distinct populations of GII-4 genetic variant norovirus in circulation in England during the 2012/13 season: one genetically similar to the NewOrleans2009 strain, and a second genetically similar to the Sydney2012 strain (Figure 2). Using this method, we designated strain types for GII-4 norovirus strains detected during the 2012/2013 season (n = 485). The majority of GII-4 strains were phylogenetically similar to the Sydney2012 strain (68.9%, n = 334/485), whilst 27.8% (n = 135/485) were genetically similar to the NewOrleans2009 strain (Table 1).

### A seasonality shift in 2012/13 was associated with the emergence of the Sydney2012 strain

The seasonal distribution of outbreaks was determined for outbreaks that met the following criteria: (i) were associated with a GII-4 norovirus strain, (ii) had been strain typed by analysis of the hypervariable P2 domain, and (iii) for which a date of outbreak had been recorded. A total of 328 outbreaks for which the date of outbreak was provided were included in this analysis: 107 outbreaks associated with NewOrleans2009 and 221 outbreaks associated with Sydney 2012.

**Figure 3:**
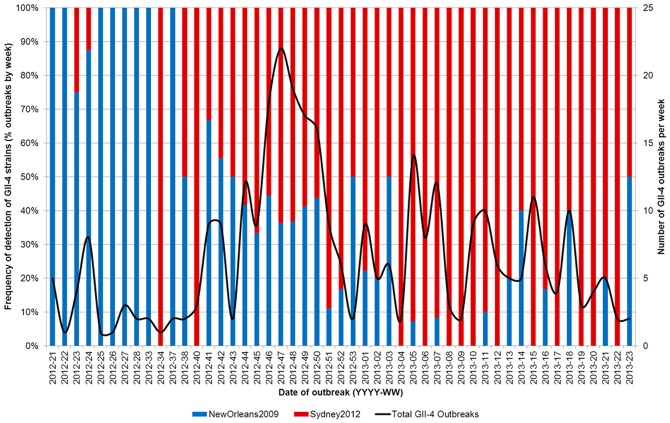
Detection of NewOrleans2009 and Sydney2012 strains associated with outbreaks (by week) in England during the 2012/13 norovirus season. Blue bars indicate outbreaks associated with NewOrleans2009. Red bars indicate outbreaks associated with Sydney2012. This figure shows data for 328 norovirus-confirmed outbreaks where GII-4 strains were associated with the outbreak, P2 domain strain typing data could be generated, and a date of outbreak was supplied.

This data showed that the peak of outbreaks occurred between week 46 2012 and week 52 2012 (November-December 2012) (Figure 3). Prior to week 46 2012, the majority of outbreaks were associated with NewOrleans2009 (48/76, 63.2% vs 28/76, 36.8%); whereas, after week 46 2012, the majority of outbreaks were associated with Sydney2012 (193/252, 76.6% vs 59/252, 23.4%) (Figure 3).

### Modelling the contribution of norovirus strains to HNORS outbreak reports in the 2012/13 season indicates an association between Sydney2012 emergence and a shift in seasonality

**Figure 4:**
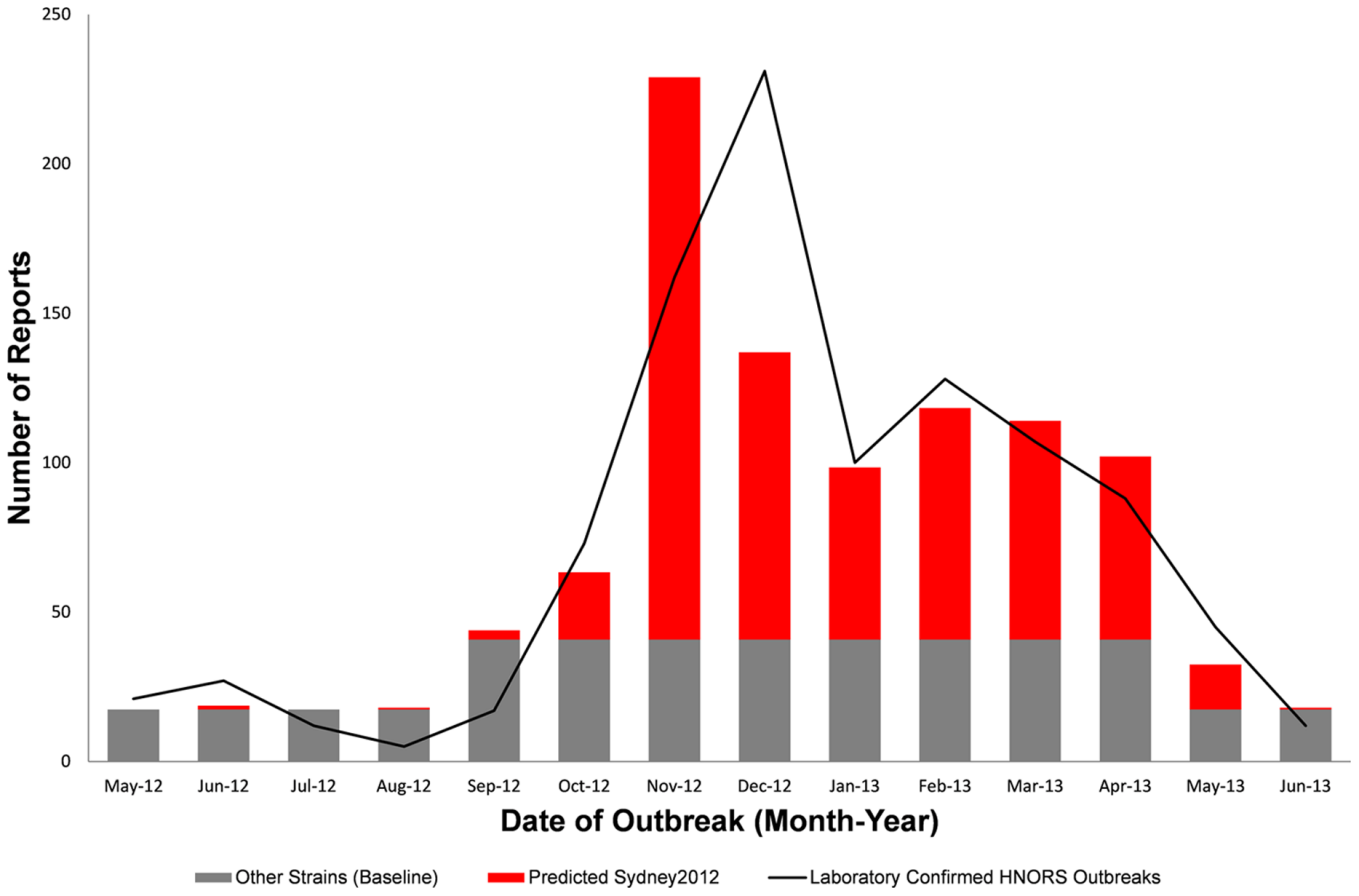
Predicted number of HNORS laboratory confirmed outbreaks associated with the Sydney2012 strain by month (May 2012–June 2013) in England. Grey bars (baseline) include all other strain types, which varies according to winter and summer as norovirus is a seasonal pathogen. Red bars indicate predicted number of HNORS outbreaks associated with Sydney2012. The black line shows the number of observed laboratory confirmed HNORS outbreaks.

Table 2 shows the comparison between the final and the initial full model. Sydney2012 was the only strain significantly associated with monthly counts of HNORS laboratory confirmed outbreaks (p<0.001). Although winter was not statistically significant (p = 0.06), the model comparisons (comparison of residual deviance) suggested that including the winter term provided a better fitting model. All other variables (strain types) were removed from the model. Observed HNORS outbreaks began to increase from September 2012, with the peak in reports observed in December 2012. The predicted contribution of Sydney2012 to HNORS outbreak reports increased from October 2012 and accounted for the majority of outbreak reports to HNORS after November 2012. By May 2013 the model suggests that Sydney2012 had declined in importance (Figure 4).

**Table 2:** Regression model results for outbreaks and norovirus strain types in England, May 2012 to June 2013.

	Initial full model		Final model	
Strain	Coefficient	p value	Coefficient	p value
**Sydney2012**	0.052	0.03	0.037	<0.001
**Other GII.4**	0.118	0.36	-	
**New Orleans**	0.002	0.94	-	
**Others**	−0.065	0.14	-	
**Winter**	0.532	0.35	0.853	0.06

### Sydney2012 is associated with distinct genetic changes in the hypervariable P2 domain

A multiple alignment (CLUSTALW) of deduced amino acid P2 domain sequences of NewOrleans2009 and Sydney2012 strains detected during the 2012/13 season was used to compare putative phenotypic differences between these two strains.

Amino acid motifs (designated by standard IUPAC single-letter amino acid codes) at two previously identified surface-exposed sites, site A (residues 296–298) and site B (residues 393–395) [11], were compared. The most commonly detected motif at site A was SRN in both NewOrleans2009 and Sydney2012 strains (96.2% and 90.1%, respectively). The most commonly detected motif at site B was STT among both NewOrleans2009 and Sydney2012 strains (90.1% and 93.3%, respectively).

**Figure 5:**
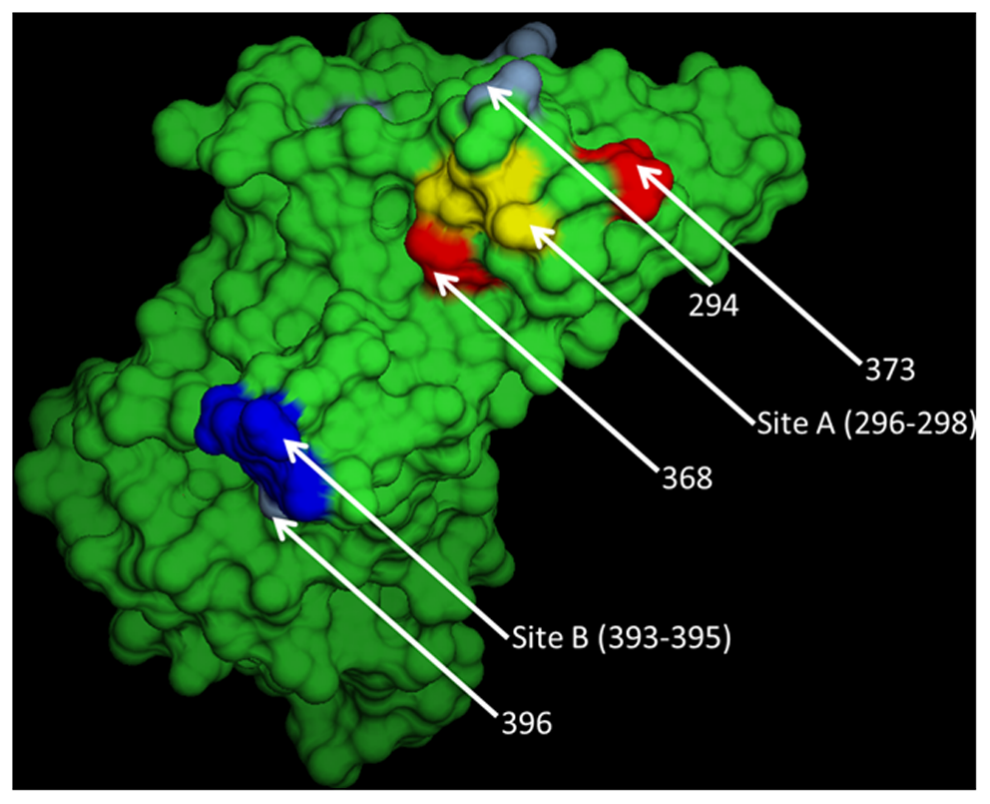
Amino acid changes in the hypervariable P2 domain associated with the Sydney2012 strain. Three-dimensional model of the GII-4 norovirus P domain [6] showing key amino acid positions on the surface of the capsid protein (grey/red). Also indicated are site A (yellow) and site B (blue) (positions 296–298 and 393–395, respectively) [11]. Details of amino acid changes are shown in Table 3.

Six amino acid positions (294, 310, 359, 368, 373 and 396) were identified from the multiple alignment that were consistently associated with Sydney2012 only (Figure 5 & Table 3). Homology modelling reveals positions 294, 368 and 373 are surface-exposed and localise around site A; and position 396 is a surface-exposed residue localised around site B (Figure 5).

**Table 3:** Characteristics of mutations at amino acid positions in the P2 domain associated with the Sydney2012.

		New Orleans 2009	Sydney 2012
	**Amino Acid**	Ser	Thr
**294**	**Characteristics**	Polar	Polar
	**Molecuar Weight (Da)**	105	119
	**Amino Acid**	Ser	Asn
**310**	**Characteristics**	Polar	Polar
	**Molecuar Weight (Da)**	105	132
	**Amino Acid**	Thr/Ser	Ala
**359**	**Characteristics**	Polar/Polar	Neutral
	**Molecuar Weight (Da)**	119/105	89
	**Amino Acid**	Ala	Glu
**368**	**Characteristics**	Neutral	Acidic
	**Molecuar Weight (Da)**	89	147
	**Amino Acid**	Asn	His
**373**	**Characteristics**	Polar	Basic
	**Molecuar Weight (Da)**	132	155
	**Amino Acid**	Pro	His
**396**	**Characteristics**	Neutral	Basic
	**Molecuar Weight (Da)**	115	155

Amino acid positions 294, 310, 359, 368, 373 and 396 were found to be associated with the Sydney2012 strain, compared with the NewOrleans2009 strain. The characteristics of the amino acid changes at these sites are shown.

## Discussion

The periodic emergence of genetically distinct GII-4 norovirus strains has been well documented [12], [13], [15]. The biological and epidemiological success of these strains is, at least in part, influenced by the fixation of mutations in the hypervariable P2 domain that affect the antigenic profile of the virus [23]–[25], which can lead to worldwide strain replacement events [26].

Following the description of the emergence of a novel GII-4 genetic strain (Sydney2012) in late 2012 [17], we followed the molecular epidemiology of this strain in England, during the 2012/13 norovirus season. Our data indicates that despite an early start to the 2012/13 norovirus season, norovirus reports also began to decline earlier than previously observed. Overall, the number of reported outbreaks to HNORS in the 2012/13 season was 9% lower than the number reported in the 2011/12 season and 9% lower than the average of the preceding three seasons. This suggests that the 2012/13 norovirus season was not associated with higher than usual activity and therefore, the emergence of Sydney2012 does not appear to have led to an increased number of outbreaks reported to HNORS in the 2012/13 season.

Using a quasi-Poisson regression generalised linear model, we modelled norovirus outbreak reports and strain-typing laboratory data from England-based surveillance systems. The objective of using the GL model was to use laboratory data (in which details of outbreaks are relatively unknown) and epidemiological data (in which strain types are unknown) in order to provide an estimate of the importance of different strains in causing outbreaks. The model suggests that Sydney2012 accounted for the majority of observed outbreaks from November 2012 to April 2013. However, whilst the emergence of Sydney2012 appears to have resulted in an early start to the 2012/13 season, it does not appear to have caused an excess number of norovirus reports in comparison to previous seasons. There are limitations to this study. The laboratory data used in the modelling was restricted to 50% of the total samples received, which could have affected the seasonal distribution of the laboratory data. Of the samples excluded, 25.1% of samples had no information on the date of the outbreak, 17.5% were negative or could not be typed, and 7.3% of samples had no information on the date of the outbreak and were negative or could not be typed. Furthermore, if these laboratory data were more likely to have included non Sydney2012 strains, the importance of Sydney2012 would be overestimated in our modelling. Likewise, if these data were more likely to have included Sydney2012, its importance would have been underestimated in the modelling. Despite these limitations there is still a considerable amount of data in comparison to other studies looking at the effect of strain type and norovirus seasonality.

Laboratory strain typing data from the 2012/13 season presented here indicates that the Sydney2012 strain could be detected as early as June 2012 (Figure 3). It is noteworthy that in week 34 2012, the Sydney2012 strain was associated with 100% of outbreaks, whilst in the weeks immediately before and after this, all outbreaks were associated with NewOrleans2009 (Figure 3), there was only one outbreak referred in week 34 2012. Furthermore, re-analysis of historic strains collected in England as part of a national strain surveillance programme indicates that the Sydney2012 strain was circulating in England as early as 2010 (Figure S1). This indicates that there was a long lag period between the first emergence and the first detection and characterisation of the Sydney2012 strain. In this period transmission networks became established through which Sydney2012 became widely disseminated internationally before its identification in 2012 [17].

Although the emergence of Sydney2012 was not associated with an increase in the number of outbreaks compared to previous seasons, a strain replacement event occurred in England during the 2012/13 norovirus season, in which the Sydney2012 strain replaced NewOrleans2009 to become the most frequently detected GII-4 norovirus strain in circulation. This suggested that the Sydney2012 strain has a biological advantage, or increased ‘fitness’ compared to the NewOrleans2009 strain. To investigate this further, we compared deduced amino acid data for the hypervariable P2 domain from GII-4 strains circulating during the 2012/13 season.

Previously, we have characterised two antigenic sites in the P2 domain that are associated with defining the antigenic profile of the virus [11], [24]. These sites (site A, positions 296–298, and site B, positions 393–395) have also been identified independently by others and shown to be significant in defining HBGA-binding and antibody blockade responses [8], [27]. Furthermore, we have shown that the predicted phenotypic changes associated with amino acid variation at these positions suggests that site A is a major epitope on the virus surface, responsible for defining the antigenic profile, whereas site B is associated with minor antigenic variation within the virus population [15].

Data from this study suggests there were no amino acid changes at these 6 positions between Sydney2012 and NewOrleans2009 strains. The previously frequently detected site A and site B motifs SRN and STT, respectively [15], were detected with high frequency among both NewOrleans2009 and Sydney2012 strains. Conservation of amino acid residues between NewOrleans2009 and Sydney2012 strains at positions 296–298 and 393–395, in part, explains why the emergence of Sydney2012 did not result in a significant increase in the number of norovirus-associated gastroenteritis outbreaks in the 2012/13 season.

The NewOrleans2009 strains have been the most frequently detected norovirus strains in England since 2009, and the site A/site B motif combination SRN/STT has been the most commonly detected among circulating strains in England since 2009 [15]. This means the majority of the population would have had recent immunological exposure to these epitopes. The conservation of these sites in Sydney2012 means the population was not entirely immunologically naïve to the Sydney2012 strain during the winter of 2012/13, and the existing immunity in the population may have been sufficient to mitigate the impact of the emergence of Sydney2012 in England.

However, Sydney2012 replaced NewOrleans2009 as the most commonly detected norovirus strain in England by November 2012; this suggests the Sydney2012 strain has a selective advantage compared to the NewOrleans2009 strains. This may be explained by amino acid changes associated specifically with the Sydney2012 strain that were detected at 6 other positions in the P2 domain (294, 310, 359, 368, 373 and 396). Positions 294, 368 and 373 are surface-exposed residues localised around the antigenic site A [11], (also identified as part of epitope A by Debbink et al, which itself overlaps with site A [8]) (Figure 5). These changes contribute to creating antigenic variation that is sufficient to increase the ‘fitness’ of Sydney2012 sufficiently for it to replace the NewOrleans2009 strain among circulating GII-4 norovirus strains, but are not sufficient to allow the virus to entirely evade existing immunity in the population without concomitant amino acid changes at sites 296–298 (and to a lesser extent 393–395). This is highlighted in a recent study by Debbink et al (2013), who also found no amino acid changes between Sydney2012 and NewOrleans2009 at positions 296–298 or 393–395, and in agreement with our data found amino acid changes at residues 294 and 368 (and 372) to be associated with the Sydney2012 strain [7]. In their paper, Debbink et al (2013) present experimental data which demonstrates that Sydney2012 is antigenically different to NewOrleans2009, and conclude that this observation is effected by amino acid changes among these residues within epitope A.

However, whilst Debbink et al (2013) demonstrate antigenic variation between Sydney2012 and NewOrleans2009 [7], surveillance and epidemiological modelling data presented here do not indicate an overall increase in the number of outbreaks in England during the last season, which would be expected following the emergence of a putative antibody escape mutant.

Together, these findings reflect the complexity in understanding immunity to this virus, and the role antibody-escape plays in the emergence of novel strains. The reduction in blockade of Sydney2012 compared to NewOrleans2009 by a monoclonal antibody described by Debbink et al (2013) demonstrates the phenotypic impact of amino acid changes in the P2 domain on the antigenic profile of the virus. However, studies using monoclonal antibodies are highly specific and do not necessarily reflect the complex polyclonal response to a natural infection.

This highlights the need for methods that allow a more rapid understanding of the phenotype of emerging genetic variants, and in particular for norovirus, the development of tools with which to interrogate the genotype-to-phenotype mapping of mutations in the virus genome. Obtaining such information is important in order to predict the likely impact of an emerging virus strain on public health. Work on influenza A virus has shown that laboratory methods which measure phenotypic differences between known genetic variants can be used to map the evolution and emergence of this important human pathogen by both genotype and phenotype [28].

Understanding the phenotypic impact of amino acid changes in the norovirus capsid is challenging. Data collected on the emergence of Sydney2012 suggests variation at positions 294, 368 and 372 and/or 373 is capable of altering the virus'phenotype, as shown in experimental work by Debbink et al (2013), and may be sufficient to induce enough ‘fitness’ change to give the Sydney2012 a selective advantage (if not complete immunological escape), as demonstrated by the strain replacement event observed worldwide during the 2012/13 season. Continued surveillance and integrated epidemiological and laboratory investigation of outbreaks and characterisation of historic and emerging strains will help improve our understanding of the dynamics of emerging norovirus strains.

## Supporting Information

Figure S1 **Neighbour-joining tree of GII-4 norovirus P2 domain amino acid sequences shows Sydney2012 was circulating in 2010, 2011, 2012 and 2013.** Sequences highlighted in red are from 2010 and 2011, indicating that the Sydney2012 strain was circulating in England as early as 2010. Strains sequenced as part of this study are labelled at the nodes as [Strain Type|OutbreakID|Date of Outbreak (YYYY-WW)]. Methods and reference strains are the same as Figure 2.
(TIF)
