# Supplementary figures and images for: Emergence of the GII-4 Norovirus Sydney2012 Strain in England, Winter 2012–2013
Source: PLoS One. 2014 Feb 13;9(2):e88978. doi: 10.1371/journal.pone.0088978 (PMC3923861; doi:10.1371/journal.pone.0088978)

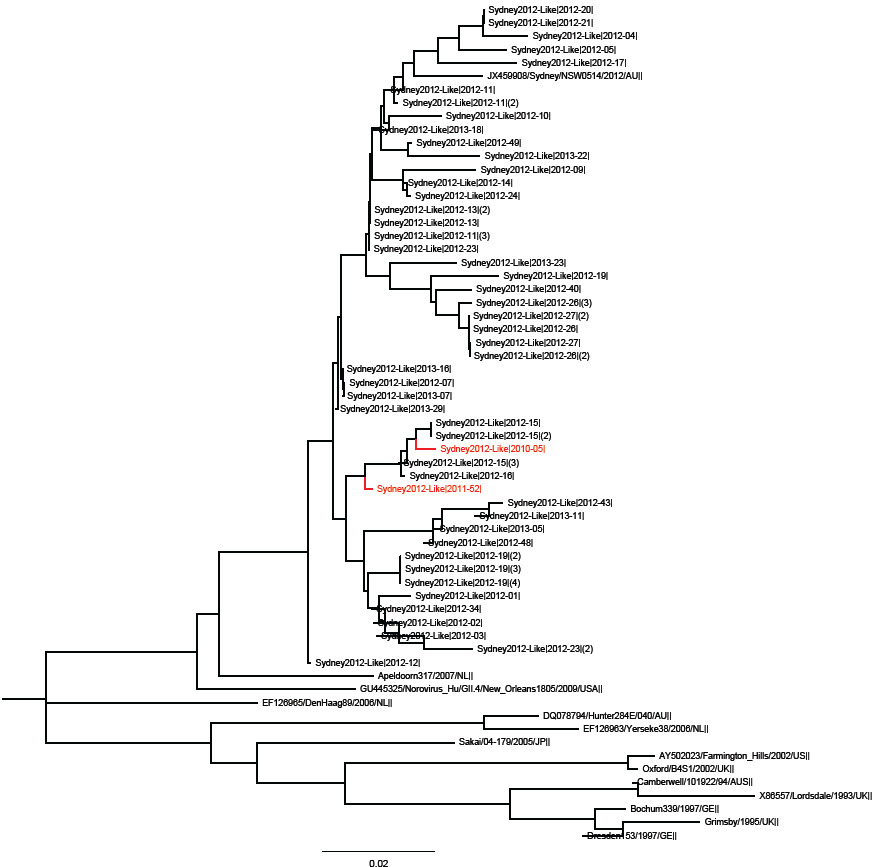

Supplement: Figure S1 — Neighbour-joining tree of GII-4 norovirus P2 domain amino acid sequences shows Sydney2012 was circulating in 2010, 2011, 2012 and 2013. Sequences highlighted in red are from 2010 and 2011, indicating that the Sydney2012 strain was circulating in England as early as 2010. Strains sequenced as part of this study are labelled at the nodes as [Strain Type|OutbreakID|Date of Outbreak (YYYY-WW)]. Methods and reference strains are the same as Figure 2. (TIF) [file pone.0088978.s001.tif]
